# Does Loneliness Have a Cost? A Population-Wide Study of the Association Between Loneliness and Healthcare Expenditure

**DOI:** 10.3389/ijph.2021.581286

**Published:** 2021-02-02

**Authors:** Rachelle Meisters, Daan Westra, Polina Putrik, Hans Bosma, Dirk Ruwaard, Maria Jansen

**Affiliations:** ^1^ Department of Health Services Research, Care and Public Health Research Institute (CAPHRI), Faculty of Health, Medicine and Life Sciences (FHML), Maastricht University, Maastricht, Netherlands; ^2^ Academic Collaborative Centre for Public Health Limburg, South Limburg Medical Health Service (GGD South Limburg), Heerlen, Netherlands; ^3^ Department of Social Medicine, Care and Public Health Research Institute (CAPHRI), Faculty of Health, Medicine and Life Sciences (FHML), Maastricht University, Maastricht, Netherlands

**Keywords:** social determinants of health, loneliness, healthcare expenditure, Netherlands, health inequalities

## Abstract

**Objectives:** Loneliness has been associated with unhealthy behavior, poorer health, and increased morbidity. However, the costs of loneliness are poorly understood.

**Methods:** Multiple sources were combined into a dataset containing a nationally representative sample (*n* = 341,376) of Dutch adults (18+). The association between loneliness and total, general practitioner (GP), specialized, pharmaceutical, and mental healthcare expenditure was tested using Poisson and Zero-inflated negative binomial models, controlling for numerous potential confounders (i.e., demographic, socioeconomic, lifestyle-related factors, self-perceived health, and psychological distress), for four age groups.

**Results:** Controlling for demographic, socioeconomic, and lifestyle-related factors, loneliness was indirectly (via poorer health) associated with higher expenditure in all categories. In fully adjusted models, it showed a direct association with higher expenditure for GP and mental healthcare (0.5 and 11.1%, respectively). The association with mental healthcare expenditure was stronger in younger than in older adults (for ages 19–40, the contribution of loneliness represented 61.8% of the overall association).

**Conclusion:** Loneliness contributes to health expenditure both directly and indirectly, particularly in younger age groups. This implies a strong financial imperative to address this issue.

## Introduction

In recent years, loneliness has become a growing public health issue. Approximately 10% of European citizens (18+) feel left out of society and the problem is greater for unemployed and low-income groups (1). While most modern Western societies perceive loneliness as a problem of old age (2), it is a growing problem in younger age groups (2, 3). Extensive research has related poor health to loneliness (4), and conversely, loneliness to unhealthy behaviors (3, 5), worse physical (6–10) and mental health (3, 10), and increased morbidity and mortality (10). In addition to the social effects of loneliness, it can thus also have a considerable impact on the ever-increasing healthcare costs of most Western countries (11). While it is imperative for well-informed policy decisions, such economic consequences of loneliness remain poorly understood.

Despite the growing awareness of loneliness as a health issue (1) and the increasing pressure on healthcare resources, research on the healthcare costs that could be attributed to loneliness is scarce. A recent review by Mihalopoulos et al. (12) identified 12 relevant studies conducted in the last 10 years. Four of these studies were cost of illness studies, which assessed various combinations of inpatient, outpatient, medical, non-medical (residential care, social services, administrative costs), or indirect costs (informal care) associated with loneliness in older adults (13–16). While most of these found that loneliness was associated with excess healthcare costs, one reported that it is associated with lower inpatient healthcare expenditure, suggesting that loneliness might act as a barrier to accessing healthcare (16). Four economic evaluation studies reported that interventions addressing loneliness may provide good value for money (12). Another five return on investment studies of loneliness interventions studied various non-monetary values, making results difficult to compare and validate (12). While some evidence thus suggests that lonely older people do have higher health care costs, little is known about other population groups (12). Furthermore, most studies focused on a limited amount of expenditure categories (e.g., only inpatient hospital care), control for a limited amount of confounding variables, and utilize relatively small samples (12).

**FIGURE 1:**
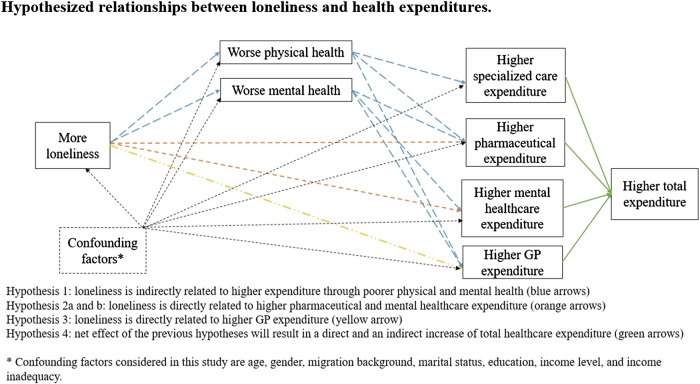
Hypothesized relationships between loneliness and health expenditures. Hypothesis 1: loneliness is indirectly related to higher expenditure through poorer physical and mental health (blue arrows). Hypothesis 2a and b: loneliness is directly related to higher pharmaceutical and mental healthcare expenditure (orange arrows). Hypothesis 3: loneliness is directly related to higher GP expenditure (yellow arrow). Hypothesis 4: net effect of the previous hypothesis will result in a direct and an indirect increase of total healthcare expenditure (green arrows). *Confounding factors considered in this study are age, gender, migration background, marital status, education, income level, and income inadequacy (The Netherlands, 2020).

The present study addresses the question “what is the relation between loneliness and healthcare expenditure?” using a large, nationally representative, sample of the general adult (18+) population. We strive to understand the association with health expenditures in the context of a broad range of potential confounding variables that are known to have an association with healthcare expenditure. As the impact of loneliness might differ between age groups and expenditure categories, we investigate the association between loneliness and general practitioner (GP), pharmaceutical, mental healthcare, specialized, and total curative healthcare expenditure in four different age groups (i.e., 19–40, 41–64, 65–80, and 81 years and older). Given the relation between loneliness and worse physical (6–10) and mental health (3, 10), we expect that loneliness is indirectly (i.e., through poorer health) associated with higher expenditure in all expenditure categories (hypothesis 1). Furthermore, we expect loneliness to be directly associated with higher a) mental healthcare and b) pharmaceutical expenditure (hypothesis 2a–b) because individuals could perceive loneliness as a mental health condition in itself, which can be treated by a mental healthcare provider or using pharmaceuticals. Additionally, lonely individuals may visit easily accessible and free-of-charge GP’s more often in search of social interactions (17). Therefore, we expect loneliness to be directly associated with higher GP expenditure (hypothesis 3). Lonely individuals of older age may lack support networks and thus seek relief for their loneliness through increased contacts with their GP’s, as opposed to their younger counterparts. Therefore, we expect differences in the associations between loneliness and GP expenditures between age groups (hypothesis 3a). For other costs categories, no a priori hypothesis was made for directions of differences by age as prior research is scarce. Explorative analyses will be undertaken. Lastly, we expect that the net effect of the previous hypotheses will result in a direct and an indirect increase of total healthcare expenditure (hypothesis 4). The hypothesized relationships in this study are visualized in Figure 1. The results of our work should provide insight to public health policy-makers who seek to understand the impact of loneliness on healthcare expenditure and economic aspects of programs targeting at alleviating loneliness.

## Methods

### Setting

We use a time-lagged design to study associations between loneliness and healthcare expenditure in the Netherlands in 2016 and 2017. Roughly, 31 billion euros were spent on curative care through compulsory health insurance schemes in the country in 2016 (18). This amounts to approximately €1800 per capita, or 4.3% of the Dutch GDP, ranking the Netherlands 13th of the 32 OECD countries on curative health expenditure (18). Dutch citizens are insured for GP services, specialized care, pharmaceuticals, and mental healthcare amongst others through compulsory basic health insurance (19).

### Data Sources and Linkage

Our dataset combines individual-level data from two sources covering the year 2016 and one source covering 2017. Firstly, we used the Health Survey of the Public Health Service 2016. It is a nationwide survey completed every 4 years by adults aged 19 years and older (*n* = 457,150). It covers various subjects including socioeconomic status (SES), social contacts, lifestyle, and general (physical and mental) health (20). It is completed by either paper and pencil, internet, telephone or face-to-face interviews. Secondly, we used data provided by Statistics Netherlands for 2016. These data included two administrative databases: the Personal Records database (PRB) and the Dutch Tax and Customs Administration data. The PRB is managed by municipalities and provided information about citizen’s age, gender, and migration background. The Dutch Tax and Customs Administration data provided income records for each citizen, for both the personal and household level. Thirdly, we used the 2017 Dutch healthcare claims dataset provided by Vektis, the healthcare information center. It is a national dataset of reimbursed individuals’ claims covered by the basic insurance package in a given year. These data have previously been used to explore associations of neighborhood disadvantage with healthcare expenditure (21). All datasets were linked via pseudonymized personal social security codes in the secured environment of Statistics Netherlands. After data linkage, our sample included 341,376 respondents.

### Measures

#### Dependent Variables

We used five dependent variables. These are 1) GP, 2) mental healthcare, 3) pharmaceutical, 4) specialized healthcare, and 5) total healthcare expenditure for the year 2017. Total healthcare expenditure is the sum of all expenditure individuals incurred under the basic health insurance plan. This includes expenditure for primary care, mental health care, pharmaceutical care, and hospital care (these four accounted for 88% of total expenditure in 2017), as well as several smaller expenditure categories such as dental-, paramedic-, obstetric-, geriatric-, cross-border care, and ambulance costs (19). In the Netherlands, GP expenditure consists of an annual enrollment fee per individual and a fee-for-service component. We use the fee-for-service component as our GP expenditure variable (i.e., expenditure associated with GP consultations). Specialized care expenditure include expenditure for specialized in-patient and outpatient clinics including in-hospital medication and excluding mental health hospitals. Pharmaceutical expenditure includes all prescription pharmaceuticals provided outside the hospital. Mental healthcare expenditure includes expenditure for basic and specialized (long and short-term) mental health services care in ambulatory or hospital settings.

#### Independent Variable

The main factor of interest in this study is loneliness, a self-reported measure based on the 11-item de Jong Gierveld scale (22), taken from the Health Survey. Work by Van Tilburg and De Jong Gierveld (23) based cutoff scores on individual’s self-assessed level on loneliness in order to keep cutoff scores more in line with individuals own perception rather than arbitrary cutoff scores. Loneliness is subsequently categorized as follows: “not lonely” (scores between 0 and 2, reference group), “somewhat lonely” (scores between three and 8), “severe loneliness” (scores of nine or 10), and “very severe loneliness” (score of 11).

#### Potential Confounders

Potential confounders included demographic, SES, lifestyle-related factors and general health measures. The demographic factors were age (19–40 as the reference group, 41–64, 65–80, and 81 years and older), gender (binary variable with male as the reference group), migration background (Dutch-born as the reference group, western migration background, and non-western migration background), and marital status (self-reported as “living together or married” as the reference group, “single,” “widowed,” or “divorced”). The three former variables were taken from the BRP, while the latter was taken from the Health Survey. The SES-variables were individuals’ highest level of completed education (higher vocational education or university degree as the reference group, secondary or middle vocational education, lower vocational education, and primary education), self-reported income adequacy (“adequate, no concerns” as the reference group, “adequate, minor concerns,” “inadequate, some concerns,” and “inadequate, major concerns”), and standardized household income based on the number of members in the household (divided it into quartiles based on the entire Dutch population, with highest quartile as reference group). The two former measures were taken from the Health Survey and the latter from the Dutch Tax Authority.

The lifestyle-related factors include body mass index (BMI), alcohol consumption, smoking behavior, and physical activity level. These were all taken from the Health Survey and are self-reported measures. BMI was categorized in “normal” (between 18.5 and 25, reference group), “underweight” (less than 18.5), “overweight” (between 25 and 30), and “obese” (over 30) (24). Alcohol consumption consists of three mutually exclusive categories; “never consuming alcohol” (reference group), “regular alcohol consumption,” or “excessive alcohol consumption” (more than 21 alcoholic beverages a week for men and more than 14 for women). The norms for alcohol consumption are based on the guidelines by the Dutch Health Council. Smoking habits were categorized as “never smoked before” (reference group), “former smoker,” and “current smoker.” Physical activity was dichotomized as being sufficient [at least 30 min of reasonably intensive activity (like walking) per day for at least 5 days a week, or a minimum of 20 min intense activity (like running) per day for at least 3 days a week, reference group] or insufficient based on the Dutch Health Council’s guidelines for sufficient physical activity (25).

General health indicators were also self-reported measures from the Health Survey: self-reported health, chronic disease, and psychological distress. Self-rated health was based on the following question; “In general, would you say your health is … .” Answer categories include “excellent,” “very good,” “good,” “fair,” and “poor.” The measure was dichotomized as either “excellent [very] good” health (reference group) or “fair or poor” health (26). Having at least one chronic disease was based on the question “Do you have one or more long-term diseases (expected duration 6 months or longer).” Answers were either no (i.e., no chronic disease) (reference group) or yes (i.e., at least one chronic disease). Psychological distress was measured with the Kessler psychological distress scale (K10) (27). The scores for these 10 questions were categorized as “none or low” (scores between 10 and 15, reference group), “moderate” (scores between 16 and 29), or “high” (scores between 30 and 50) psychological distress.

Lastly, as mode of completing the survey (internet, paper and pencil, telephone or face-to-face interviews) can impact the answers (28), it was adjusted for in each model.

### Statistical Analyses

The survey sample was weighted to represent the overall Dutch population, based on age, gender, ethnicity and urbanization levels. The regression analyses accounted for survey design.

Healthcare expenditure data are often skewed and/or contain excessive zeros, requiring specific analytical approaches (29). Vuong and Zero-Inflated Poisson likelihood-ratio tests guided the choice of final model (30). We consequently performed our analyses using Poisson (for total expenditure) and zero-inflated negative binomial (ZINB) regressions (for GP, specialized, pharmaceutical, and mental healthcare expenditure). In ZINB regressions, the output is twofold. The first part provides the Incidence Rate Ratio (IRR) for non-zero expenditure, assuming a Poisson distribution. Second, the inflated part of the output represents the odds of incurring zero expenditure (vs. any expenditure). For this study, the IRR represents the expected expenditure incurred for a lonely person (somewhat, severe or very severely lonely), divided by the expected expenditure incurred for a non-lonely person, accounting for covariates.

For each expenditure category, 6 models were computed by adding new covariates at each step. That is, expenditure were first modeled with loneliness as the only predictor in model 1. Next, demographic variables were added in model 2, SES variables were added in model 3, lifestyle variables were added in model 4, self-perceived health variables (self-rated health and chronic disease) were added in model 5, and the psychological distress variable was added in model 6, which represents the fully adjusted model. The mode of completing the survey was included in all models 1–6. To determine the need for subgroup analyses, we tested for interaction effects between loneliness and age in the different expenditure categories. Lastly, to estimate expenditure of loneliness, marginal expenditure estimates were obtained from the models with all covariates held constant at their average value. These were than extrapolated to the entire Dutch population for the year 2017. The significance level was set at alpha = 5%. All analyses were performed in Stata 15 (31).

## Results

### Descriptive Statistics

Table 1 reports the descriptive statistics. The mean (SD) age was 59.3 (16.9) years and 52.7% of the sample was female. The prevalence of loneliness was 41.8%, with 33.5% of the respondents experiencing some loneliness, 5.4% severe, and 2.9% very severe loneliness. Chronic diseases were reported at least once for 39.3% of the sample, and 26.1% reported their health as (very) bad or fair. Over half of the population reported none or low psychological distress (60.7%), 34.8% reported moderate, and 4.5% high psychological distress. Loneliness was prevalent in all age groups, however more common in older age groups. The prevalence of loneliness was 34.8% in 19–40 year-olds, 39.7% in 41–64 year-olds, 43.7% in 65–80 year-olds and 57.4% for respondents of 81 years and older, see Table 2.

**TABLE 1:** Sample characteristics (*n* = 341,376) (The Netherlands, 2020).

Sample characteristics		*n* (%)	Sample characteristics		*n* (%)
Age^a^	19–40	55,817 (16.4%)	Physical activity^b^	Insufficient	96,417 (28.2%)
	41–64	118,814 (34.8%)		Sufficient	244,959 (71.8%)
	65–80	143,231 (42.0%)	BMI^b^	Underweight (<18, 5)	4,260 (1.2%)
	81+	23,514 (6.9%)		Normal (18, 5–25)	155,082 (45.4%)
Gender^a^	Male	161,576 (47.3%)		Overweight (25–30)	131,625 (38.6%)
	Female	179,800 (52.7%)		Obese (30>)	50,409 (14.8%)
Migration background^a^	Dutch-born	300,426 (88.0%)	Alcohol consumption^b^	Never	33,799 (9.9%)
	Western migration background	28,697 (8.4%)		Regular consumption	280,475 (82.2%)
	Non-western migration background	12,253 (3.6%)		Excessive	27,102 (7.9%)
Marital status^a^	Married/co-habitant	248,688 (72.8%)	Smoking^b^	Never smoked	138,456 (40.6%)
	Single	36,338 (10.6%)		Former smoker	147,920 (43.3%)
	Widowed	23,533 (6.9%)		Current smoker	55,000 (16.1%)
	Divorced	32,817 (9.6%)	Chronic disease^b^	None	207,262 (60.7%)
Education^b^	Primary school	19,897 (5.8%)		At least one	134,114 (39.3%)
	Lower vocational	106,023 (31.1%)	Self-rated health^b^	Excellent, (very) good	252,118 (73.8%)
	Middle vocational/secondary	107,937 (31.6%)		Fair, poor	89,258 (26.2%)
	Higher vocational/university	107,519 (31.5%)	Psychological distress^b^	None or low	207,079 (60.6%)
Household income quartile^a^	0–25%	43,471 (12.7%)		Moderate	1198,853 (34.8%)
	26–50%	86,582 (25.4%)		High	15,444 (4.6%)
	51–75%	99,759 (29.2%)	Loneliness^b^	Not lonely	198,705 (58.2%)
	76–100%	111,564 (32.7%)		Somewhat lonely	114,428 (33.5%)
Self-reported income adequacy^b^	Inadequate, major concerns	9,690 (2.8%)		Severely lonely	18,393 (5.4%)
	Inadequate, some concerns	34,973 (10.2%)		Very severely lonely	9,850 (2.9%)
	Adequate, minor concerns	117,764 (34.5%)	Completing	Paper and pencil	149,630 (43.8%)
	Adequate, no concerns	178,949 (52.4%)	Survey	Internet	191,249 (56.0%)
				Face-to-face	337 (0.1%)
				Telephone	160 (0.05%)

BMI, body mass index; GP, general practitioner.

a Registry data variables.

b Self-reported variables extracted from Health Survey.

**TABLE 2:** Prevalence loneliness across age groups N (%) (The Netherlands, 2020).

	19–40	41–64	65–80	81+
Not lonely	36,383 (65.2%)	71,665 (60.3%)	80,639 (56.3%)	10,018 (42.6%)
Somewhat lonely	15,123 (27.1%)	36,985 (31.1%)	51,611 (36.0%)	10,709 (45.5%)
Severely lonely	2,870 (5.1%)	6,365 (5.4%)	7,228 (5.0%)	1,930 (8.2%)
Very severely lonely	1,441 (2.6%)	3,799 (3.2%)	3,753 (2.6%)	857 (3.6%)

### Assoiations between loneliness and expenditure categories

Table 3 reports the associations of loneliness with different categories of healthcare expenditure. In models 1 to 4, loneliness is associated with higher expenditures, albeit with smaller (and in specialized care some non-significant) IRRs in models 4, partially confirming hypothesis 4. After controlling for all potential confounders (model 6, Table 3), loneliness was still directly associated with increased mental healthcare expenditure, confirming hypothesis 2a. That is, the IRR for loneliness categories ranged between 1.17 (1.04–1.33) and 1.31 (1.08; 1.58), indicating higher expenditure in mental healthcare for lonely people compared to non-lonely people. Model 6 also indicates a small direct increase of GP expenditure for individuals reporting higher levels of loneliness [1.08 (1.04–1.13)], in line with hypothesis 3. However, the association of very severe loneliness with pharmaceutical expenditure was no longer statistically significant [1.00 (0.85–1.18)] in model 6, rejecting hypothesis 2b. The association between very severe loneliness and specialized care expenditure was negative in model 6 [i.e., IRR of 0.88 (0.80–0.97)].

**TABLE 3:** Associations of loneliness with total healthcare, GP, specialized, pharmaceutical, and mental healthcare expenditure (The Netherlands, 2020).

		1. Loneliness	2. Demographic and loneliness	3. Demographic, SES, and loneliness	4. demographic, SES, lifestyle, and loneliness	5. Demographic, SES, lifestyle, self-perceived health, and loneliness	6. Total: demographic, SES, lifestyle, self-perceived health, psychological distress, and loneliness
		IRR 95% (CI)	IRR 95% (CI)	IRR 95% (CI)	IRR 95% (CI)	IRR 95% (CI)	IRR 95% (CI)
Total expenditure	Not lonely	Ref	Ref	Ref	Ref	Ref	Ref
	Somewhat lonely	**1.38 (1.33–1.42)**	**1.24 (1.20–1.28)**	**1.17 (1.13–1.20)**	**1.14 (1.10–1.18)**	1.00 (0.97–1.03)	**0.96 (0.93–0.99)**
	Severely lonely	**1.81 (1.72–1.92)**	**1.66 (1.57–1.76)**	**1.45 (1.37–1.53)**	**1.38 (1.31–1.46)**	1.04 (0.99–1.10)	0.96 (0.91–1.02)
	Very severely lonely	**2.10 (1.95–2.25)**	**1.93 (1.80–2.08)**	**1.58 (1.47–1.70)**	**1.50 (1.39–1.61)**	1.05 (0.99–1.12)	0.94 (0.87–1.01)
GP Expenditure	Not lonely	Ref	Ref	Ref	Ref	Ref	Ref
	Somewhat lonely	**1.24 (1.22–1.25)**	**1.18 (1.16–1.19)**	**1.12 (1.11–1.14)**	**1.12 (1.10–1.13)**	**1.06 (1.05–1.08)**	**1.02 (1.01–1.04)**
	Severely lonely	**1.53 (1.49–1.57)**	**1.45 (1.41–1.49)**	**1.32 (1.28–1.36)**	**1.30 (1.26–1.33)**	**1.16 (1.13–1.20)**	**1.07 (1.04–1.10)**
	Very severely lonely	**1.72 (1.66–1.79)**	**1.65 (1.58–1.72)**	**1.43 (1.38–1.50)**	**1.41 (1.35–1.47)**	**1.22 (1.17–1.27)**	**1.08 (1.04–1.13)**
Specialized care Expenditure	Not lonely	Ref	Ref	Ref	Ref	Ref	Ref
	Somewhat lonely	**1.11 (1.06–1.16)**	1.05 (1.00–1.11)	1.01 (0.96–1.07)	1.01 (0.96–1.06)	**0.93 (0.88–0.98)**	**0.94 (0.90–0.98)**
	Severely lonely	**1.24 (1.15–1.34)**	**1.25 (1.14–1.37)**	**1.15 (1.05–1.26)**	**1.12 (1.03–1.22)**	0.93 (0.85–1.00)	0.94 (0.87–1.02)
	Very severely lonely	**1.26 (1.14–1.38)**	**1.26 (1.13–1.39)**	**1.12 (1.01–1.24)**	1.09 (0.98–1.20)	**0.87 (0.79–0.95)**	**0.88 (0.80–0.97)**
Pharmaceutical Expenditure	Not lonely	Ref	Ref	Ref	Ref	Ref	Ref
	Somewhat lonely	**1.39 (1.30–1.48)**	**1.30 (1.21–1.40)**	**1.21 (1.12–1.30)**	**1.18 (1.10–1.27)**	1.02 (0.97–1.07)	1.02 (0.96–1.07)
	Severely lonely	**1.76 (1.58–1.99)**	**1.76 (1.52–2.04)**	**1.52 (1.31–1.76)**	**1.47 (1.26–1.71)**	1.05 (0.94–1.18)	1.04 (0.94–1.16)
	Very severely lonely	**1.90 (1.71–2.12)**	**1.77 (1.58–1.99)**	**1.43 (1.28–1.60)**	**1.38 (1.23–1.55)**	1.01 (0.87–1.18)	1.00 (0.85–1.18)
Mental healthcare Expenditure	Not lonely	Ref	Ref	Ref	Ref	Ref	Ref
	Somewhat lonely	**1.40 (1.23–1.60)**	**1.35 (1.19–1.53)**	**1.29 (1.14–1.47)**	**1.26 (1.12–1.43)**	**1.21 (1.08–1.37)**	**1.17 (1.04–1.33)**
	Severely lonely	**1.50 (1.26–1.79)**	**1.47 (1.25–1.73)**	**1.36 (1.17–1.57)**	**1.29 (1.12–1.48)**	**1.20 (1.04–1.37)**	1.09 (0.95–1.26)
	Very severely lonely	**1.85 (1.49–2.29)**	**1.82 (1.44–2.31)**	**1.59 (1.34–1.97)**	**1.62 (1.33–1.97)**	**1.47 (1.22–1.78)**	**1.31 (1.08–1.58)**
	Inflated part reported in Supplementary Appendix A4						

Coefficients with *p* < 0.05 are in bold. GP, General practitioner; IRR, Incidence rate ratio; CI, confidence interval; SES, Socioeconomic status. Results from Poisson and Zero-inflated negative binomial regressions (n = 342,095). Inflated part reported in Supplementary Appendix A4.

### Marginal Expenditure of Loneliness

Table 4 reports the point estimate of marginal spending of loneliness (in million €) for the different healthcare categories in 2016, with the corresponding 95% confidence interval, and the percentage of overall annual spending in each category. In the fully adjusted model (model 6), loneliness was associated with higher expenditure for mental and GP care (confirming hypothesis 2a and 3) but not for other expenditure categories. For GP expenditure, loneliness was associated with 5.8 million euros (4.5–7.1), or 0.8% of the total annual GP spending (Table 4). For mental healthcare, loneliness was associated with 340.2 million euros (314.7–365.8), or 10.3% of the annual mental healthcare spending. For total healthcare and specialized care expenditure, loneliness was associated with 1.0% [435.4 million euros (−494.8 to −376.1)], and 2.0% [449.8 million (−474.3 to −425.2)] fewer spending, rejecting hypothesis 4.

**TABLE 4:** Marginal effects of loneliness for healthcare expenditure, extrapolated to entire Dutch 18+ population (The Netherlands, 2020).

Million € [95% CI] (% category spending 2017)					
	Total expenditure	GP expenditure	Specialized care expenditure	Pharmaceutical expenditure	Mental healthcare expenditure
A. model 6: fully adjusted model					
Somewhat lonely	−315.1 [−299.0 to −331.2] (−0.7)	3.3 [3.0–3.5] (0.5)	−314.4 [−289.0 to −339.9] (−1.4)	13.7 [−4.4 to 31.8] (0.3)	243.4 [235.9–250.9] (7.4)
Severely lonely	−63.0 [−100.4 to −25.6] (−0.1)	1.7 [1.2–2.3] (0.2)	−69.2 [−97.2 to −41.3] (−0.3)	6.0 [−7.7 to 19.8] (0.0)	55.2 [48.5–62.0] (1.7)
Very severely lonely	−57.3 [−95.3 to −19.3] (−0.1)	0.9 [0.3–1.4] (0.1)	−66.2 [−88.2 to −44.2] (−0.3)	−1.2 [−14.3 to 11.9] (0.1)	41.6 [30.3–52.8] (1.3)
Total	−435.4 [−494.7 to −376.1] (−1.0)	5.8 [4.5–7.1] (0.8)	−449.9. [−474.3 to −425.4] (−2.0)	18.5 [−26.4 to 63.4] (0.4)	340.2 [314.7–365.8] (10.3)
B. model 3: basic model + SES					
Somewhat lonely	1,273.2 [1,248.1–1,298.5] (3.0)	17.8 [19.2–20.3] (2.8)	200.2 [194.0–206.4] (0.9)	201.9 [175.1–228.8] (4.3)	497.1 [455.9–538.3] (15.1)
Severely lonely	647.6 [578.0–717.3] (1.5)	9.7 [8.9–10.4] (1.4)	190.8 [141.1–240.2] (0.8)	95.6 [66.4–124.7] (2.1)	212.7 [183.4–242.1] (6.5)
Very severely lonely	484.5 [416.0–553.0] (1.1)	7.3 [6.6–8.1] (1.0)	112.1 [74.6–149.7] (0.5)	49.8 [38.8–60.9] (1.1)	179.4 [143.0–215.8] (5.4)
Total	2,405.4 [2,242.0–2,568.8] (5.6)	36.7 [34.7–38.7] (5.2)	503.1 [422.0–584.1] (2.2)	347.4 [280.3–414.4] (7.5)	889.3 [782.3–996.2] (27.0)
C. model 2: basic model					
Somewhat lonely	1,797.0 [1,758.6–1,835.3] (4.2)	27.6 [26.9–28.3] (3.9)	450.9 [452.7–450.1] (2.0)	289.0 [255.3–322.7] (6.2)	569.1 [521.1–617.2] (17.3)
Severely lonely	921.7 [837.5–1,005.8] (2.2)	13.3 [12.5–14.2] (1.9)	305.7 [247.6–364.0] (1.4)	138.2 [103.5–172.9] (3.0)	274.0 [231.2–316.8] (8.3)
Very severely lonely	744.0 [657.8–830.2] (1.7)	10.8 [9.8–11.8] (1.5)	208.8 [164.0–253.8] (0.9)	85.5 [70.2–101.0] (1.8)	253.9 [191.8–316.2] (7.7)
Total	3,462 [3,253.8–3,671.4] (8.1)	51.8 [49.3–54.3] (7.3)	965.4 [864.3–1,068.0] (4.3)	512.7 [429.0–596.6] (11.0)	1,097.0 [943.9–1,250.1] (33.3)

GP, general practitioner; CI, confidence interval; SES, socioeconomic status. A. model 6 is the most extensive model, which includes loneliness, demographic, SES, lifestyle, self-perceived health and psychological distress. B. model 3 includes loneliness, demographic, and SES factors. C. model 2 is the basic model, which includes loneliness and demographic factors. Results from Poisson and Zero-inflated negative binomial regressions (*n* = 342,095).

### Subgroup Analyses

**FIGURE 2:**
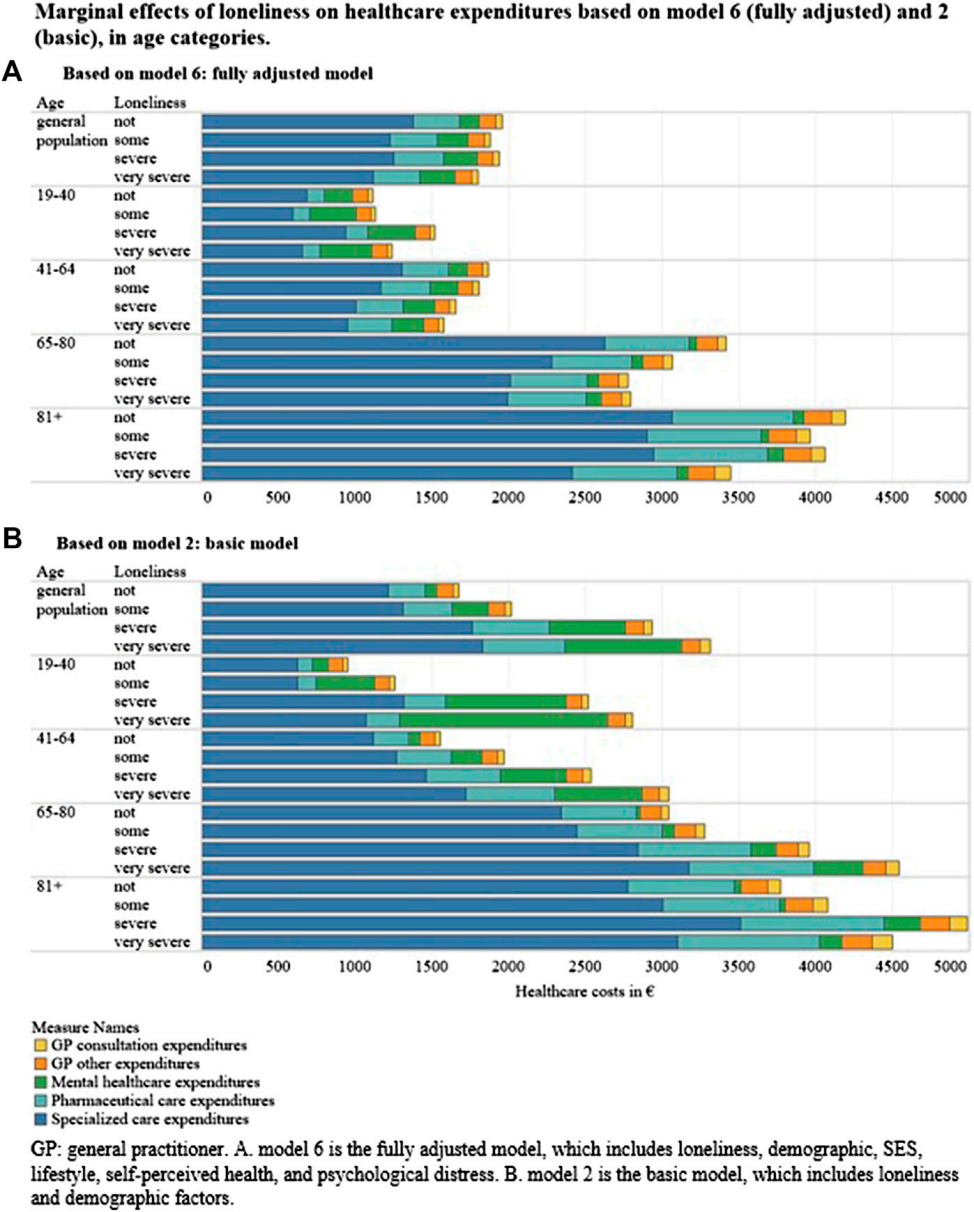
Marginal effects of loneliness on healthcare expenditures based on model 6 (fully adjusted) and 2 (basic), in age categories. **(A)** Based on model 6: fully adjusted model. **(B)** Based on model 2: basic model. GP, general practitioner. A) model 6 is fully adjusted model, which includes loneliness, demographic, SES, lifestyle, self-perceived health, and psychological distress. B) model 2 is the basic model, which includes loneliness and demographic factors (The Netherlands, 2020).

The interaction effects between age and loneliness were significant for total, pharmaceutical and mental healthcare, indicating a different association between loneliness and expenditure across age groups for these categories. Figure 2 (Supplementary Appendix) visualizes the spending patterns incurred per expenditure category for non-lonely, somewhat lonely, severely lonely, and very severely lonely individuals in the entire sample as well as in each age group (i.e., 19–40, 41–64, 65–80, and 81+). The corresponding IRR’s and CI’s of loneliness are reported in the Supplementary Appendix Table A1. Figure 2 and Supplementary Appendix Table A1 show that in the fully adjusted model (model 6) expenditure for adults over 40 (total and pharmaceutical) tend to be lower with increasing loneliness. Furthermore, age and loneliness do not have a significant interaction effect for GP expenditure, rejecting hypothesis 3a. Conversely, for the youngest age group (19–40), total expenditure is higher for severely lonely respondents compared to those who do not report loneliness. For mental healthcare, expenditure were even higher with very severe loneliness (IRR of 1.83 [1.34; 2.50]). In percentages, 6.3% of mental healthcare expenditure can be attributed to loneliness for age group 19–40, 3.1% in 41–64 year-olds, 0.7% in 65–80 year-olds, and 0.1% fewer healthcare spending in 81+ year-olds, in fully adjusted models. This represents 61.8, 30.0, 6.9, and 1.3% of the overall contribution of loneliness and increased mental healthcare expenditure, per age group, respectively.

## Discussion

This study assessed the impact of loneliness on different types of healthcare expenditure, controlling for a range of individual demographic factors, socio-economic, lifestyle, and health indicators. The study is based on a linked, large dataset resulting in a nationally representative sample of the Dutch adult population (*n* = 341,376). Firstly, our results reveal that loneliness is associated with higher indirect spending in all expenditure categories (i.e., models 1–4), in line with hypothesis 1. However, as the model was further adjusted for self-perceived health and psychological distress, the positive association between loneliness and expenditure reversed. The pattern of higher spending for lonely individuals namely only holds for mental healthcare and GP expenditure, confirming hypothesis 2a and 3. Contrarily, the association is non-significant for pharmaceutical and total expenditure (hypotheses 2b and 4) and is even reversed (i.e., lonely individuals incur fewer expenditure) for specialized healthcare expenditure.

The reduction (and in some cases reversing) of the association between loneliness and expenditure across the models suggests that the relationship between loneliness and expenditure might be mediated by self-perceived health and psychological distress. While this finding is in line with an extensive body of research that relates loneliness to worse physical (6–10) and mental health (3, 10), it complicates determining the total amount of (healthcare related) expenditure associated with loneliness, and hence rejecting or confirming hypothesis 4. As our results indicate, loneliness may be associated with an indirect increase of 3.5 billion euros (8.1%) of total healthcare expenditure in the simplest estimation, or a direct decrease of 435.4 million euros (1.0%) of total healthcare expenditure in the fully adjusted model. Lower expenditure associated with more loneliness are particularly apparent in specialized care. This could be explained by avoidance of care by lonely people compared to non-lonely people (16). Since specialized care represents a large part of total healthcare expenditure, the net results of all hypotheses result in lower total healthcare expenditure (hypothesis 4).

Further research, preferably with longitudinal designs, is required to clarify the underlying causal or complex mechanisms of loneliness and increased or decreased expenditure in all categories as well as between potential confounders. Longitudinal study designs could provide insight into effects of chronic loneliness over time on health care consumption. Furthermore, longitudinal designs could unravel underlying reverse causal mechanisms between poor health and loneliness. We hypothesized that loneliness leads to poorer mental and physical health. Alternatively however, poorer health may also lead to increased loneliness (4). In particular, reversed causality between poor mental health and increased loneliness may arise due to decreasing social support and resources of mentally ill individuals (4). In contrast, further research might find poorer physical health (i.e., accidents or severe illnesses) associated with less loneliness if treatments and social support are intensified. These potentially alternative pathways cannot be disentangled in a cross-sectional study, warranting further longitudinal research.

Nevertheless, our results do show a robust association between loneliness and higher mental healthcare expenditure. Even in the fully adjusted model, loneliness is associated with 10% (i.e., 340 million euros) additional mental healthcare expenditure annually (hypothesis 2a). This implies that (new) policies or societal programs targeted at combatting loneliness may have the potential to significantly reduce healthcare expenditure, particularly in mental healthcare. As shown in models one to six, loneliness may affect healthcare expenditure through different pathways (i.e., via worsened self-perceived health and psychological distress). Both economic and health aspects of loneliness should be considered in the development of new public health policies and societal programs in practice. Policies and programs combatting loneliness may even become more relevant in times of pandemic outbreaks and social restrictions as seen in the recent COVID-19 outbreak.

Secondly, our study is the first to reveal distinct associations of loneliness and healthcare expenditure across various age groups. While most policies and research associates loneliness with older age (2) and we expected healthcare expenditure to be higher for older age groups, our findings clearly indicate that severe loneliness is associated with relatively higher expenditure in younger adults (i.e., in aged 19–40) compared to older age groups, particularly for mental healthcare. This is consistent with researchers reporting that younger generations perceive higher levels of stress in today’s more individualistic, high-performance society (32). Programs to address loneliness should target beyond older aged populations, and potential savings in (mental) healthcare expenditure should be considered in economic evaluations of programs.

## Limitations

This study is not without limitations. First, some subgroups of the general population are under-represented in the Health Survey dataset. Examples include people of lower SES, with poorer health (33), or institutionalized citizens. However, survey design has taken this into account by oversampling low SES groups and the data were weighted for underrepresentation to mitigate these effects. Nevertheless, the associations for mental healthcare may still be underestimated as institutionalized citizens were not included. Second, this study produced cost estimates for the hypothesized relationships in the conceptual model. In view of the alternative mechanisms mentioned above, these estimates should only be interpreted very cautiously as an estimate of the healthcare related cost of loneliness. Simultaneously, we hope, the estimates indicate that loneliness not only comes with socioemotional costs, but also with financial costs. Third, more research is needed to further validate the cutoff points suggested by van Tilburg and de Jong-Gierveld (23).

## Conclusion

Loneliness is associated with higher healthcare expenditure in all types of curative healthcare services independent of demographic-, socioeconomic- and lifestyle factors. For mental healthcare and GP spending, loneliness was associated with higher expenditure independent of demographic-, socioeconomic-, lifestyle factors, self-perceived health, and psychological distress. In the other categories, the association of loneliness and increased expenditure may be indirect (i.e., mediated in particular by self-perceived health and psychological distress). Furthermore, contrary to common perceptions of loneliness as an old-age problem, our results show that it plays a larger role in explaining healthcare expenditure in younger adults than it does in older adults. Societal programs targeting at loneliness thus have the potential to generate significant savings in healthcare expenditure, especially in mental healthcare and for younger people.

## Data Availability Statement

The dataset was provided by Statistics Netherlands and the Dutch Public Health Services. Requests to access these datasets should be directed to Statistics Netherlands, microdata@cbs.nl. Results are based on calculations by researchers from Maastricht University using non-public microdata from Statistics Netherlands.

## Ethics Statement

The studies involving human participants were reviewed and approved by Ethical Review Committee of the Faculty of Health, Medicine and Life sciences of Maastricht University (FHML-REC/2019/025). The patients/participants provided their written informed consent to participate in this study.

## Author Contributions

RM, DW, PP, HB, DR, and MJ contributed to the design of the work. RM performed analyses. RM, DW, and PP drafted the first draft which was critically reviewed and approved for submission by all authors.

## Conflict of Interest

The authors declare that the research was conducted in the absence of any commercial or financial relationships that could be construed as a potential conflict of interest.
